# Carcinoma of the Larynx

**Published:** 1890-09-27

**Authors:** 


### CARCINOMA OF THE LARYNX.

One of the best papers contributed to the surgical section of the International Congress at Berlin was by Mr. Henry Butlin, assistant surgeon at St. Bartholomew's Hospital, "on radical operations for the cure of intrinsic carcinoma of the larynx." The author treated especially of the great principle which should underlie the selection of cases for operation. Mr. Butlin applies the term intrinsic "to carcinomas arising in the ventricular band, the ventricle, the true cord, and the parts below the cord," while he considers as extrinsic carcinomas which have their origin in the epiglottis, ary-epigolottic fold, the intra-arytenoid fold, and the pyriform sinus. Intrinsic carcinomas do not affect the lymphatic glands early, while extrinsic carcinomas usually affect the glands at a very early period. Extrinsic carcinoma is, therefore, a dire disease, running a rapid course, and seldom or never cured, or even checked by operation. Intrinsic carcinoma is a less formidable malady. Mr. Butlin, therefore, suggested, some years ago, that the operation of excision of the larynx should be practised only in cases of intrinsic carcinoma, in which the disease is still limited to the interior of the larynx. With the assistance of Dr. Kanthack, 102 operations for intrinsic carcinoma have been collected. These 102 operations comprise 28 cases of thyrotomy, with the removal of diseased parts in the interior of the larynx. Three of these died from the results of the operation; in 13 cases the disease recurred; eight patients were well at various periods, but sufficient time has not elapsed to allow of their being regarded as cured; one patient died at the end of 13 months, without recurrence of the disease. In three cases the operation may be regarded as wholly successful, for one of the patients died four years after at the age of 67; the second was well at the end of eight years, and the third at at the end of twenty years. There were 23 cases of partial, generally half, excision of the larynx. Of these, seven died from the operation, six suffered from recurrence, five recovered, and were well at various periods within three years, and one died at the end of two two years and a-half without recurrence. Four patients may be claimed as cured, for three were well at the end of three years and a-half, four years, and four years and a-half after the operation; and a fourth died at the end of five years of cerebral apoplexy. There were 51 complete excisions of the larynx, the operation proving fatal in 16. The disease recurred in 17; four patients were well at the end of various short periods; and six died of other causes. It may be claimed that eight patients were cured, for seven were well at the end of three years and a-half, and the others at the termination of varying periods extending to nine years, one, however, dying after four years from another cause, without recurrence of carcinoma. Some of these patients were employed in avocations which may be regarded as laborious — one man in a riding-school, and a woman as a laundress. Of deaths due to the various operations there were, therefore, 26—pneumonia or septic poisoning being the direct cause in the majority of instances. Mr. Butlin only regards those patients as cured, who were free from disease three years after the operation. The total results, therefore, so far as cure is concerned, are that 15 patients were alive and free from disease, or died of some other disease than cancer, at periods of from three to 20 years after the operation, a result which, Mr. Butlin thinks, compares favourably with those afforded by operation for malignant disease of some other parts of the body. Dr. Melville Wassermann collected 118 cases of complete excision of the larynx for carcinoma. These operations were performed for both intrinsic and extrinsic carcinoma. In these 118 cases there were eight complete successes. With reference to the choice of operation in individual cases, Mr. Butlin urges that the smallest operation, consistent with the widest excision of the disease, and with the removal of a wide area of the surrounding tissues should be performed. To ascertain the extent and situation of the disease it is essential that the larynx should be widely opened. It will usually be found that the disease occupies a wider space than shown by the laryngoscope. Removal of the cartilages and bones, which form the larynx, is in most cases of intrinsic carcinoma unnecessary. It suffices to cut away, or scrape away, with a Volkmann's spoon all the softened parts. At first sight Mr. Butlin's tables appear to show that the results of partial and complete excision are better than those afforded by thyrotomy. But the mortality of partial and complete excision is more than twice that due to thyrotomy. "The life of a person from whom the whole larynx has been removed is very precarious." He is more liable to bronchitis, to pneumonia, and to accident. All this must be taken into account when considering the relative value of these operations, and it must be admitted that the comfort of the lives of patients from whom the whole larynx has been removed is often seriously affected.

